# Medical and Biomedical Students’ Perspective on Digital Health and Its Integration in Medical Curricula: Recent and Future Views

**DOI:** 10.3390/ijerph22081193

**Published:** 2025-07-30

**Authors:** Srijit Das, Nazik Ahmed, Issa Al Rahbi, Yamamh Al-Jubori, Rawan Al Busaidi, Aya Al Harbi, Mohammed Al Tobi, Halima Albalushi

**Affiliations:** 1 Department of Human and Clinical Anatomy, College of Medicine and Health Sciences, Sultan Qaboos University, Muscat 123, Oman; s.das@squ.edu.om; 2 College of Medicine and Health Sciences, Sultan Qaboos University, Muscat 123, Oman; nazik2001tayfour@gmail.com (N.A.); erahbi4@gmail.com (I.A.R.); aljuboriyamamh@gmail.com (Y.A.-J.); rawanhalbusaidi@gmail.com (R.A.B.); ayaalharbi2002@gmail.com (A.A.H.); altoubim11@gmail.com (M.A.T.)

**Keywords:** digital health, e-Health, students, medical, biomedical, curricula

## Abstract

The incorporation of digital health into the medical curricula is becoming more important to better prepare doctors in the future. Digital health comprises a wide range of tools such as electronic health records, health information technology, telemedicine, telehealth, mobile health applications, wearable devices, artificial intelligence, and virtual reality. The present study aimed to explore the medical and biomedical students’ perspectives on the integration of digital health in medical curricula. A cross-sectional study was conducted on the medical and biomedical undergraduate students at the College of Medicine and Health Sciences at Sultan Qaboos University. Data was collected using a self-administered questionnaire. The response rate was 37%. The majority of respondents were in the MD (Doctor of Medicine) program (84.4%), while 29 students (15.6%) were from the BMS (Biomedical Sciences) program. A total of 55.38% agreed that they were familiar with the term ‘e-Health’. Additionally, 143 individuals (76.88%) reported being aware of the definition of e-Health. Specifically, 69 individuals (37.10%) utilize e-Health technologies every other week, 20 individuals (10.75%) reported using them daily, while 44 individuals (23.66%) indicated that they never used such technologies. Despite having several benefits, challenges exist in integrating digital health into the medical curriculum. There is a need to overcome the lack of infrastructure, existing educational materials, and digital health topics. In conclusion, embedding digital health into medical curricula is certainly beneficial for creating a digitally competent healthcare workforce that could help in better data storage, help in diagnosis, aid in patient consultation from a distance, and advise on medications, thereby leading to improved patient care which is a key public health priority.

## 1. Introduction

Digital health transforms healthcare by leveraging technology to enhance patient care, efficiency, and accessibility. The World Health Organization (WHO) defines digital health as “the field of knowledge and practice associated with the development and use of digital technologies to improve health” [1]. This includes mobile health (mHealth), electronic health records (EHRs), telemedicine, wearable devices, electronic prescribing systems (EPS), and web-based health services (WHS) [2]. These technologies have proven beneficial in disease prevention, diagnosis, and management, while expanding access to health information and health promotion services [3].

The COVID-19 pandemic accelerated the adoption of digital health, with tools such as video consultations, mobile apps for contact tracing, and wearable devices like smartwatches and oxygen monitors becoming essential for patient monitoring and communication [4]. College students represent a key demographic that can benefit from digital health and are more likely to utilize internet for health-related matters. University students, particularly young adults, represent a key demographic that can benefit from digital health due to their significant health risks, and their widespread access to internet-based healthcare solutions [5]. A study found that 34.9% of undergraduate students used at least one health-related mobile application, while 3.9% utilized wearable health devices, highlighting gaps in adoption based on gender, field of study, and academic year [5]. Digital health applications can be tailored to student’s needs [6].

Beyond student populations, healthcare practitioners also stand to benefit from digital health integration. Digital tools can reduce phone call loads, decrease waiting room congestion, and improve communication precision. There is the advantage of communicating with patients in a remote area, saving the time needed for visits and waiting and being more cost effective [7]. A study in Europe showed that using digital tools resulted in a decrease in phone load, increase in efficiency, less crowding in the waiting rooms, and more precise communication [8]. Published studies have shared strong views on incorporating the implementation of digital tools into medical education and stressed the importance of the use of the latest digital tools [9]. Another published study reported the facts that digital healthcare is important in clinical care but it has not been well-represented in medical curriculum [10]. A survey of 451 European medical students found that while 40.6% were comfortable working in a digital healthcare system, while 53.2% reported insufficient knowledge due to inadequate education on the subject [6].

Digital health technologies, ranging from telemedicine and mobile health applications to artificial intelligence and electronic health records, are transforming the landscape of modern healthcare and public health. These tools play a critical role in addressing key public health priorities, including expanding access to care, improving chronic disease management, and responding to global health emergencies such as the COVID-19 pandemic. As future physicians, medical students represent a crucial link in the adoption and diffusion of digital health innovations. Their perceptions, attitudes, and preparedness will significantly influence the integration of digital tools into clinical practice and, by extension, the effectiveness of public health systems. Understanding medical students’ perspectives on digital health is therefore essential not only for medical education reform but also for ensuring a digitally competent workforce capable of supporting public health advancement in the digital era.

It is essential to assess the awareness, perspectives, and readiness of medical and biomedical students toward digital health. Understanding their attitudes toward digital health technologies and their integration into medical curricula will provide insights into how best to equip future healthcare professionals for a digitally driven healthcare landscape. This study aims to explore the knowledge and awareness, influencing factors, and perceptions about the future of digital health among Omani undergraduate medical and biomedical students at Sultan Qaboos University. By examining their readiness to embrace digital healthcare tools, this research will contribute to the ongoing discussion on the necessity of digital health education in medical training.

## 2. Materials and Methods

### 2.1. Study Design and Participants

This was a cross-sectional study conducted between August 2023 and November 2023 on medical and biomedical undergraduate students at the College of Medicine and Health Sciences at Sultan Qaboos University (SQU). Foundation program students and students transferred from the College of Medicine to another college were excluded. The target sample size was 500. The response rate was 37% (186/500). Informed consent was implied by the participant’s completion and submission of the survey. At the beginning of the survey, participants were informed that their responses would remain confidential, that participation was optional, and that they could decline to answer any question or withdraw at any point before submission. By proceeding to complete and submit the survey, participants indicated their understanding of the study’s purpose and their consent to participate.

### 2.2. Ethical Considerations

This study was approved by the Medical Research Ethics Committee, College of Medicine and Health Sciences, Sultan Qaboos University (REF.NO.SQU. EC/265/2022).

### 2.3. Data Collection

The questionnaire included items on a 5-point Likert scale. The questionnaire included (i) socio-demographic information about the students; (ii) general questions on e-Health; (iii) dimensions of e-Health; and (iv) perceptions of the future of e-Health and e-Health in medical curriculum in Oman. The validated questionnaire was adapted from a published article (“Perceptions of Digital Health Education Among European Medical Students: Mixed Methods Survey” by Machleid et al. [6]). Permission was obtained from the corresponding author and the questionnaire was modified to accommodate the situation in Oman.

The initial questionnaire was piloted by distributing it to a small number of participants in the target sample. The analysis and feedback received through the pilot questionnaire were used to produce the final questionnaire. The final questionnaire was distributed electronically through Google Forms, and the participants were invited to fill it out through email.

### 2.4. Data Analysis

The data analysis was performed using Statistical Package for the Social Sciences software (SPSS^®^ version 29). Descriptive statistics present the demographic data and questionnaire items presented using frequencies, bars, and pie charts. Inferential analysis was conducted using the Chi-square (χ^2^) test to assess associations between categorical variables. This non-parametric test was applied to determine whether there were statistically significant differences in the distribution of responses across different groups (e.g., year of study, gender). A *p*-value of less than 0.05 was considered statistically significant.

## 3. Results

### 3.1. Demographic Data

The total number of respondents was 186, and 96 individuals (51.61%) were males, while 90 individuals (48.39%) were females. The mean age of the respondents was approximately 20.9 years, with an age range from 18 to 25 years. The most prevalent age group was 21 years, comprising 63 individuals (33.87%).

The majority of respondents are enrolled in the MD (Doctor of Medicine) program, with 157 students (84.4%) participating, while 29 students (15.6%) were enrolled in the BMS (Biomedical Sciences) program. Regarding the year of study, the most significant representation was MD: Phase 2 with 105 students (56.45%), followed by MD: Phase 1 with 28 students (15.05%), and MD: Phase 3 with 24 students (12.90%). The distributions for the other years in the BMS and MD programs showed fewer participants.

### 3.2. General Questions on e-Health

The definition of e-Health refers to the use of information and communication technologies in the field of healthcare and this includes components such as electronic health records, telemedicine and health information system. The majority of respondents, specifically 55.38%, agreed that they were familiar with the term ‘e-Health,’ which suggests a notable familiarity with the concept. The participants were asked about their familiarity with the term e-Health; 58% reported that they agreed and 16% strongly agreed that they were familiar with the term e-Health. However, 8% disagreed with the statement. Additionally, 143 individuals (76.88%) reported being aware of the definition of “e-Health”, indicating a general awareness of its relevance in the healthcare sector. No significant correlation was observed between the gender of the medical students and familiarity with e-Health (*p* = 0.315) or between the year of study reflected by the phase of the study and familiarity with e-Health (*p* = 0.341) (Table 1).

Furthermore, 149 individuals (80.11%) indicated that they understood the differences between e-Health and e-learning, acknowledging that e-Health focuses on healthcare services, while e-learning pertains to educational delivery through digital means. Further analysis of the respondents’ responses to the familiarity of the difference between e-Health and e-learning revealed no significant correlation between the year of study and familiarity with the difference between e-learning and e-Health (*p* = 0.416). Majority of the students reported that they knew the difference between the two terms regardless of their year of study in the biomedical sciences program or the MD program (Table 2).

The data regarding the frequency of using e-Health technologies indicates that most students engage with these tools regularly. Specifically, 69 individuals (37.10%) utilize e-Health technologies every other week, while 20 individuals (10.75%) report using them daily. Additionally, 44 individuals (23.66%) indicated never using such technologies. This reflects significant engagement with digital health tools among respondents and underscores the importance of e-Health in their daily lives. (Figure S1).

### 3.3. Perceptions of e-Health Technologies

Respondents were asked to evaluate the use of three categories of digital health technologies: mHealth, Telehealth, and big data. The mHealth or mobile health refers to the medical and public health practice supported by mobile devices, such as mobile phones, patient monitoring devices, and other wireless devices. Telehealth, also termed as telemedicine refers to the use of telecommunications and virtual technology to deliver healthcare outside of traditional healthcare facilities. The big data refers to data of a very large size, typically to the extent that its manipulation and management present significant logistical challenges. Responses were categorized into five levels: Mainly advantages, More advantages, Undecided, More disadvantages, and Mainly disadvantages.

Regarding mHealth, 29.3% of respondents indicated “Mainly advantages,” while 49.3% reported “More advantages”. A smaller proportion (15.3%) were “Undecided,” and only 2% and 4% reported “More disadvantages” and “Mainly disadvantages,” respectively.

Regarding Telehealth, 25.3% perceived “Mainly advantages,” and 52% indicated “More advantages.” Similar to mHealth, a notable proportion (14.7%) were “Undecided,” with lower levels of dissatisfaction (2.7% reported “More disadvantages”).

**Figure 1:**
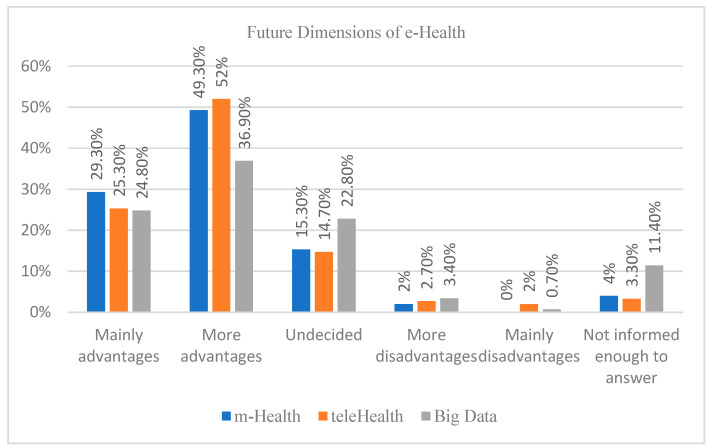
Medical and biomedical students’ perspectives regarding mHealth, telehealth, and big data use. Data presented as percentages.

Concerning big data, 24.8% noted “Mainly advantages,” while 36.9% reported “More advantages.” The percentage of undecided respondents was slightly higher (22.8%), and dissatisfaction remained minimal (3.4% and 2.1% for “More disadvantages” and “Mainly disadvantages,” respectively) (Figure 1).

### 3.4. Responsibilities for Managing Health Data

Opinions regarding responsibilities for managing health data were evaluated using two key statements: “Healthcare professionals should be responsible for managing health data,” and “Patients should manage their own health data.”

For the first statement, 26.7% of respondents “Strongly Agreed,” and 54.7% “Agreed,” indicating significant support for healthcare professionals taking primary responsibility. Meanwhile, 10.7% were “Undecided,” and a small percentage disagreed (6.7%) or strongly disagreed (1.2%).

In contrast, for the second statement, only 10% “Strongly Agreed,” and 20.7% “Agreed” with patients managing their own data. A larger percentage (24.7%) were “Undecided,” while 32.7% “Disagreed,” and 11.9% “Strongly Disagreed.” (Figure S2).

### 3.5. Access to Patients’ Health Data

Respondents were asked their opinions on which third parties should have access to patients’ health data. The majority of the 150 participants who answered this question (78.7%, n = 118) agreed that doctors and hospital staff should have access. Other notable groups included health insurance companies (37.3%, n = 56), universities/other research institutions (32.7%, n = 49), and legal/state authorities (26%, n = 39).

**Figure 2:**
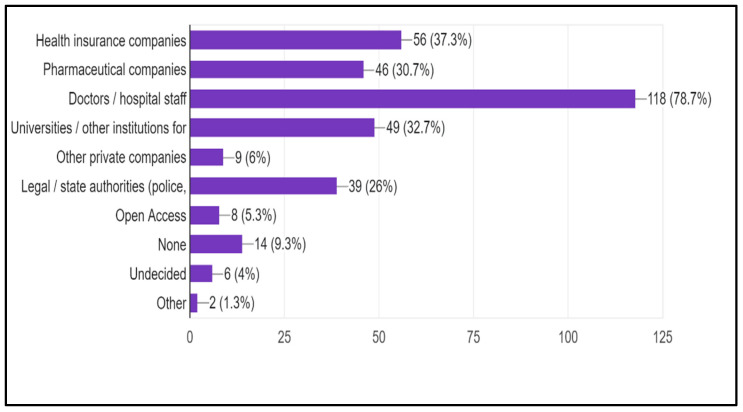
Medical and biomedical students’ opinions on which third parties should have access to patients’ health data. The percentage is calculated as the number of students who choose a specific party over the total number of those who responded to this question (150). Data presented beside each bar represents the number (percentage).

In contrast, lower agreement was observed for pharmaceutical companies (30.7%, n = 46), other private companies (6%, n = 9), and open access platforms (5.3%, n = 8). A small portion of respondents indicated that no third parties should have access (9.3%, n = 14), were undecided (4%, n = 6), or selected other (1.3%, n = 2) (Figure 2).

### 3.6. Management of Data

There were different opinions about patients’ ability to manage their data; 31% of the participants agreed, but 42% disagreed on the ability of patients to manage their own data. Most participants (34.8%) think doctors and hospital staff should access patients’ data, and 15% believe that health insurance companies should have access. This highlights the need for a balanced approach to data access, ensuring both patient privacy and healthcare efficiency, a concern that should be at the forefront of our discussions on e-Health. The study reveals divergent perspectives on the control of e-Health data, with the majority advocating for national-level regulation (50.4%), followed by preferences for regional (29.3%) and hospital-level control (19.5%).

### 3.7. The Future of e-Health

When asked about the potential role that e-Health could play in the future of medicine, respondents showed confidence in their expectations. It was generally agreed upon by those who participated in the survey that digital health can dramatically improve not only the delivery of healthcare but also the outcomes for patients. Around 76% believed that e-Health would revolutionize medicine.

### 3.8. e-Health in the Medical Curriculum

The comments made by the participants suggested that there is a definite need for more structured and thorough training, even though the majority of the participants assessed their e-Health competencies as “Good” and voiced support for the statement, “I would like e-Health to be more implemented in the medical curriculum.” While some students were able to take advantage of digital literacy classes and specific e-Health technologies, the majority of students believed that the curriculum that was currently being offered was insufficient. Further analysis revealed that there is no significant correlation between the year of study and the students’ views regarding the implementation of digital health in the medical curricula (*p* = 0.408). The majority of the students supported the implementation of e-health in the medical curricula (Table 3).

### 3.9. e-Health Literacy

It was found that the majority of respondents, i.e., 68%, rated their level of e-Health literacy as Good or above. Notably, 40% of respondents selected “Good,” 18% selected “Very good,” and 10% selected “Acceptable.” There is also a general desire for new e-Health topics to be introduced into the curriculum of medical schools. This desire is common. There were various degrees of confidence among those who participated in the survey, particularly with regard to the actual implementation of advanced digital tools and the level of understanding of these tools. Fifty-five percent of the students were in agreement or strongly agreed that they felt prepared, while 25% were still hesitant, and 20% were in disagreement or strongly disagreed.

## 4. Discussion

Participants were asked if they were aware of the definition of e-Health and the difference between it and e-learning. More than half of the participants agreed or strongly agreed that they were aware of the term e-Health. Among these participants, 38% reported using digital health technology every other week, and 21% never used it. This finding is not only intriguing but also novel, warranting further investigation. It aligns with a previous European Medical Students’ Association study, which found that 53.0% of European students agreed they were familiar with the e-Health term. However, 60.8% of them never use it, indicating a significant gap between familiarity and usage [6].

Our study was in agreement with a previous study, which may be attributed to the unique circumstances in Oman. Here, we have a high usage of digital health apps, especially during the COVID-19 pandemic. This indicates a promising potential for e-Health in our country. Additionally, the government has introduced a new healthcare application (SHIFA) for all citizens, which could have influenced the participants’ views and usage patterns, further underlining the positive trajectory of digital health in Oman. It is reassuring to note that the majority of the participants, 81%, demonstrated a clear understanding of the difference between e-Health and e-learning. This indicates a solid foundation of knowledge among the participants, which is crucial for effectively implementing digital health technologies.

As mentioned previously, regarding views toward the use of the different dimensions of e-Health, we can conclude that the participants have a more advantageous view towards the use of e-Health (79%) followed by telehealth (77%) and big data, with only 61% of the participants having advantageous views towards it. This can be explained by the fact that 24% of the participants were undecided, and 11% did not feel they had enough information to answer about their views on the future use of big data in medicine. So, there is not much information about big data compared to mHealth or telehealth [5].

A study in France showed that 34.9% of the participants had at least one mobile health application, which is one of the mHealth dimensions [5]. It is a challenge how to educate the professionals in healthcare to work in an environment of digital tools and there is need to incorporate topics related to digital health into the curriculum [11]. Another published study showed that mHealth apps are being used more frequently and have proven to be beneficial for individuals with chronic conditions, helping in behavior change for self-management and improving the communication between patients and providers [12]. So, the frequency of the usage of mHealth compared to telehealth and big data leads to a more advantageous view for future use in medicine. Big data has been shown to improve laboratory care and result in better patient care [13]. Big data has also proved to be beneficial for the diagnosis of chronic diseases and predicting outcomes of various diseases [14].

The present study revealed divergent perspectives on the control of e-Health data, with a majority advocating for national-level regulation (50.4%), followed by preferences for regional (29.3%) and hospital-level control (19.5%). Each level of control has its unique benefits and challenges. However, the real challenge lies with policymakers who must reconcile these views to develop effective regulatory frameworks. This balancing act involves maintaining autonomy while promoting standardization, fostering collaboration, and ensuring ethical data stewardship in healthcare. In general, most participants are very optimistic about the future implementation of information and communication technologies in medical practice and research.

An earlier study has highlighted the importance of personal health records (PHRs) and mHealth apps as vital tools for patient engagement [15]. The authors of the same study also stated how the PHR could reduce the inconvenience related to insulin management, medications, and allergies [15].

When asked about the potential role that e-Health could play in the future of medicine, respondents showed confidence in their expectations. It was generally agreed upon by those who participated in the survey that e-Health has the ability to dramatically improve not only the delivery of healthcare but also the outcomes for patients. The findings indicate that there is considerable support for the establishment of national laws on e-Health technologies. The purpose of these regulations is to ensure that they are applied in the same manner throughout all healthcare systems. Respondents stated that they had a good attitude towards the application of communication and information technology in medical practice. This is because it can improve access to healthcare resources and optimize efficiency in clinical processes, as demonstrated by the respondents’ positive attitude.

The fact that the e-Health courses given were frequently brief and that they typically lasted for less than 5 h indicates that comprehensive training is deficient. An extensive range of topics, ranging from fundamental digital literacy to more specialized fields such as telemedicine, artificial intelligence, data security, health informatics, and the utilization of medical applications, was suggested for inclusion. In their discussion, participants emphasized the importance of future healthcare workers having a solid understanding of emerging technologies such as artificial intelligence (AI) and digital health. Studies have highlighted how AI-enhanced electronic records could be beneficial in primary care [16]. Use of AI for e-Health could also increase diagnostic precision and better therapeutic outcomes [17].

It was observed that the majority of respondents (68%) rated their level of e-Health literacy as Good or above. Notably, 40% of respondents selected “Good,” 18% selected “Very good,” and 10% selected “Acceptable.” There is also a general desire for new e-Health topics to be introduced into the curriculum of medical schools. This desire is common. There were various degrees of confidence among those who participated in the survey, particularly with regard to the actual implementation of advanced digital tools and the level of understanding of these tools. Fifty-five percent of the students were in agreement or strongly agreed that they felt prepared, while 25% were still hesitant, and 20% were in disagreement or strongly disagreed. These patterns were confirmed by qualitative remarks, as several respondents brought up the necessity of further hands-on training in clinical applications. This finding is in line with another previous study, which showed that 20% of respondents were unaware of service availability [18]. Based on these findings, it is possible to infer that there is a strong desire for more comprehensive e-Health education to be included in the medical curriculum. Earlier studies reported positive outcomes between e-Health literacy and health-related behaviors [19].

It should be acknowledged that, in the present-day scenario, there are some barriers to digital health integration. Medical schools in Oman have started using digital health tools in recent times. Our institution was originally meant for 50 students, but gradually the number of students admitted increased. Thus, there was an urgent need for adequate infrastructure for the increased number of MD students which could not be fulfilled easily. Also, it was during the COVID-19 that the need increased and the construction of infrastructure was suddenly given a thrust. The program needed the information technology expertise of staff who were trained overseas, and thus took time to implement. The integration of the old information technology with the latest one took considerable time. During recent times, the institution developed and upgraded 3D virtual laboratories and made it compulsory for all teaching to cover certain aspects of e-learning to enhance the integration of digital health into the curriculum and ensure that students are exposed to some of its modalities.

Interestingly, there are a few reports published from the Middle East region regarding the barriers to telemedicine. An earlier study showed doctor and patient resistance, lack of infrastructure, insufficient funding, inferior system quality, and lack of adequate information technology training to be the main causes of barriers to telemedicine [20]. Another study in India found that a lack of proper funding, obstruction to change, lack of policy, investment issues, and the lack of a collaborative approach were the main challenges [21]. Another study found that issues such as inadequate training, cultural differences, and privacy matters were the main challenges for digital health in South Asia [22]. A study in Malaysia found that the most common barrier to using digital health was the dilemma on which apps to use [23]. An interesting study in Singapore found that the students refrained from using mHealth as they had a fear of stigma and were worried about confidentiality [24].

The findings of the present study underscore the critical importance of integrating digital health education into medical training to prepare future physicians for technology-driven healthcare delivery. While students generally acknowledged the relevance of digital tools in clinical care and public health, many reported insufficient exposure or confidence in using these technologies. These gaps pose a potential threat to the successful implementation of digital health interventions, which are increasingly central to public health strategies aimed at improving health equity, system efficiency, and chronic disease surveillance. If left unaddressed, this disconnect between awareness and readiness may hinder the healthcare system’s ability to meet evolving population health needs, especially in underserved or resource-limited contexts. Bridging this gap through targeted curriculum development and experiential learning is a necessary step toward building a resilient, digitally literate medical workforce aligned with public health imperatives.

### Limitations and Recommendations

This research has many limitations, for example, the low response rate due to the length of the questionnaire and that the questionnaire was distributed to the participants during the semester, alongside their exams and regular workload, which may also affect their seriousness in filling out the questionnaire. We admit that the response rate was modest, and this could have affected representativeness, especially when it comes to the biomedical sciences students’ perspectives. In the institution where this study was conducted, there are more medical students than biomedical students. The usual yearly intake for the MD program is approximately 100 students while it is 40 for the biomedical program. This limitation may affect the generalization of the findings we have in this study. Furthermore, in our study, the MD students comprised Phases 1,2 and 3. It should be mentioned that Phase 3 students have more clinical encounters compared to Phase 1 and 2 and even BMS students. Clinical teachings often use digital tools more frequently. Thus, it is common to have MD students more acquainted with digital tools compared to BMS students and the results may be accepted with caution.

Including medical students from another university in Oman may give a clearer view of the digital health perspective among the students in Oman. Doctors from the hospitals may also be involved to explore the graduate medical students’ perspectives toward digital technology’s role in medicine.

## 5. Conclusions

In conclusion, most medical and biomedical students at Sultan Qaboos University were familiar with the definition of digital health. However, the majority of them use it less frequently than anticipated. Wearable and mobile applications (m-Health) are the most widely known and used by students. At the same time, big data is the least well-known and used. Most participants agreed with the importance of the future use of digital health in medicine. The national level may be the appropriate level for digital health control, per the participants. Overall, the participants had a favorable view of the future implementation of technology in medical practice.

The present study highlights that medical students recognize the importance of digital health but require greater institutional support and training to confidently engage with these tools. Given the growing reliance on digital technologies in addressing public health challenges, from managing non-communicable diseases to enhancing pandemic responses, equipping future healthcare providers with digital competencies is both an educational and public health priority. Medical education reform that includes structured digital health training is essential to ensure that tomorrow’s physicians are not only clinically competent but also capable of advancing public health outcomes in an increasingly digital world.

Oman Vision 2020 may provide an excellent platform for the incorporation of digital health. Generative artificial intelligence tools could be used in the field of digital health to train young medicos. There is a need to incorporate digital health into the medical curriculum as it may help in better utilization of technology to facilitate innovation and the comprehensive training of young medical personnel. Personalized healthcare strategies, consultations from a distance, tracking of metrics related to the patient healthcare, and diagnostic outcomes could benefit from the incorporation of digital health into the medical curriculum. Digital health technology could help to provide high-quality patient care and improve patient outcomes.

## Figures and Tables

**Table 1:** The distribution of medical students according to their phase of study in the MD program, gender and their responses regarding their familiarity with the term e-Health.

Response to the Statement “I am Familiar with the Term of ‘e-Health”	Gender	MD		
		**Phase 1**	**Phase 2**	**Phase 3**
Agree and strongly agree	Male	5	42	9
	Female	12	39	8
Disagree and strongly disagree	Male	3	4	1
	Female	1	1	3
Undecided	Male	4	11	3
	Female	3	8	0

**Table 2:** The distribution of medical and biomedical students in different years of study and their response to the question asking them about their knowledge about the difference between e-Health and e-learning.

		Knowing the Difference Between e-Learning and e-Health		Total
		No	Yes	
Year of study	MD phase 1	3	25	28
	MD phase 2	25	80	105
	MD phase 3	5	19	24
	BMS year 1	1	2	3
	BMS year 2	1	1	2
	BMS year 3	1	8	9
	BMS year 4	1	14	15
Total		37	149	186

**Table 3:** The distribution of medical and biomedical students in different years of study and their responses regarding the implementation of digital health in the medical curricula.

		Students’ Response to the Statement (“I Would Like e-Health to be more Implemented in the Medical Curriculum”)			Total
		Agree and Strongly Agree	Disagree and Strongly Disagree	Undecided	
Year of study	MD phase 1	22	1	5	28
	MD phase 2	75	8	22	105
	MD phase 3	23	0	1	24
	BMS year 1	3	0	0	3
	BMS year 2	1	0	1	2
	BMS year 3	8	0	1	9
	BMS year 4	9	1	5	15
Total		141	10	35	186

## Data Availability

Data is available upon request.

## Supplementary Materials

The following supporting information can be downloaded at: https://www.mdpi.com/article/10.3390/ijerph22081193/s1, Figure S1: The frequency of using e-health in daily life.; Figure S2: The breakdown of subjects who ‘Strongly agreed, Agreed, were Undecided, Disagreed, StronglY agreed, and not informed enough to answer.
