# Supplementary material for: Medical and Biomedical Students’ Perspective on Digital Health and Its Integration in Medical Curricula: Recent and Future Views
Source: Int J Environ Res Public Health. 2025 Jul 30;22(8):1193. doi: 10.3390/ijerph22081193 (PMC12385753; doi:10.3390/ijerph22081193)
Supplement: Supplementary file 1 [file ijerph-22-01193-s001.zip › ijerph-3734360-supplementary.pdf]

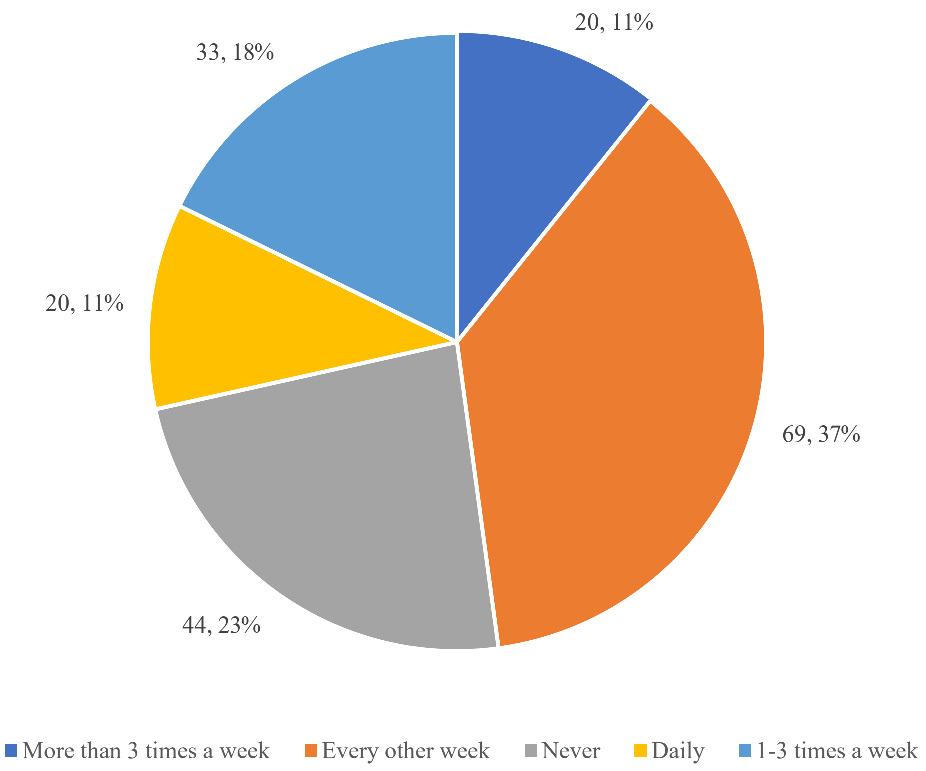


Figure S1 showing the frequency of using e-health in daily life.

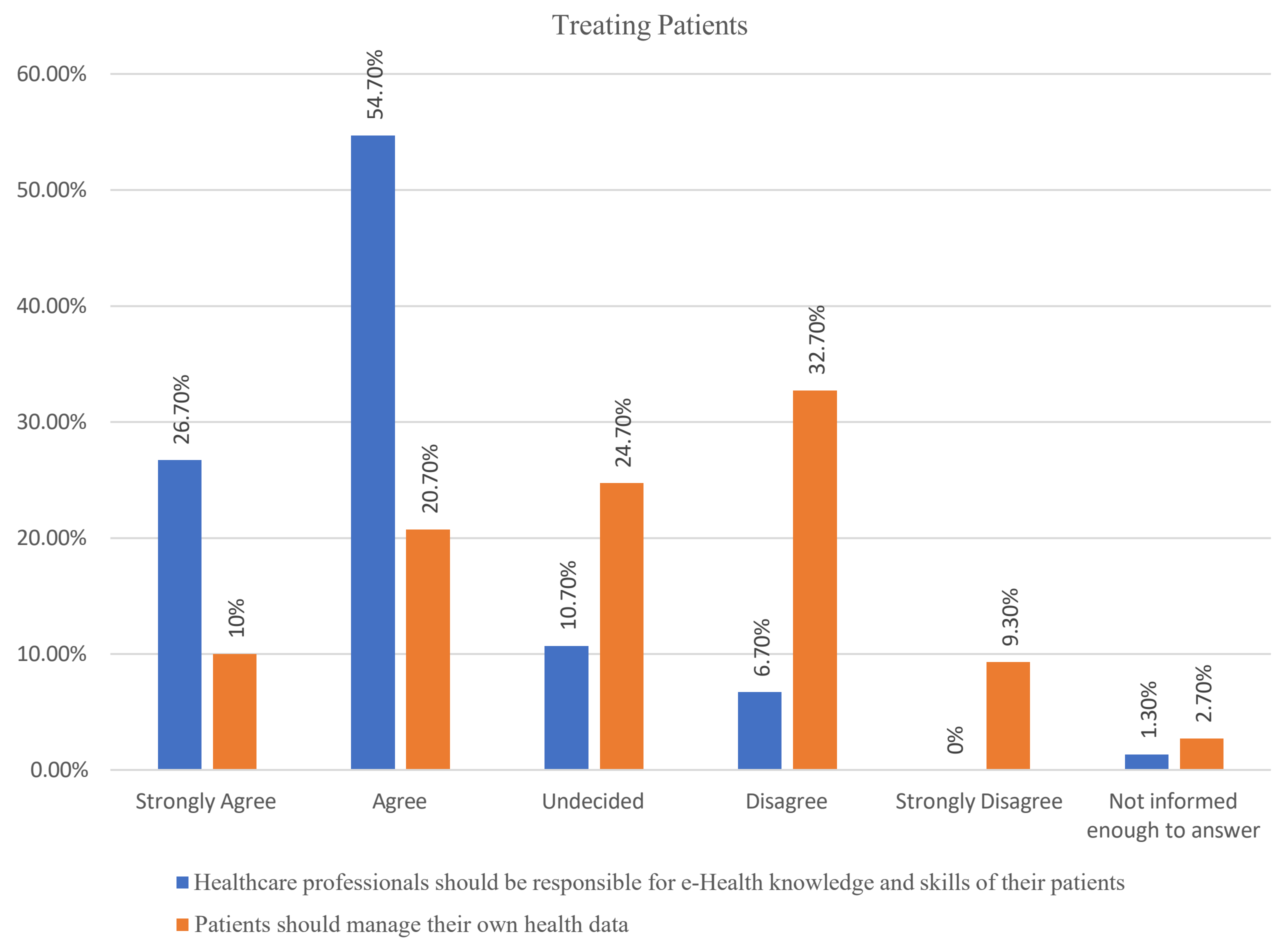


Figure S2 showing breakdown of subjects who 'Strongly agreed, Agreed, were Undecided, Disagreed, Strongly disagreed, and not informed enough to answer.
